# CCI-007, a novel small molecule with cytotoxic activity against infant leukemia with *MLL* rearrangements

**DOI:** 10.18632/oncotarget.10022

**Published:** 2016-06-14

**Authors:** Klaartje Somers, Daria A. Chudakova, Shiloh M.C. Middlemiss, Victoria W. Wen, Molly Clifton, Alan Kwek, Bing Liu, Chelsea Mayoh, Angelika Bongers, Mawar Karsa, Sukey Pan, Sarah Cruikshank, Marissa Scandlyn, Wendi Hoang, Toshihiko Imamura, Ursula R. Kees, Andrei V. Gudkov, Olga B. Chernova, Michelle Haber, Murray D. Norris, Michelle J. Henderson

**Affiliations:** ^1^ Children's Cancer Institute, University of New South Wales, Sydney, New South Wales, Australia; ^2^ Department of Pediatrics, Kyoto Prefectural University of Medicine, Kyoto, Japan; ^3^ Telethon Kids Institute, University of Western Australia, Perth, Western Australia, Australia; ^4^ Department of Cell Stress Biology, Roswell Park Cancer Institute, Buffalo, NY, USA; ^5^ Oncotartis, Inc., Buffalo, NY, USA

**Keywords:** MLL-rearranged leukemia, CALM-AF10 leukemia, HOXA9/MEIS pathway, small molecule, apoptosis

## Abstract

There is an urgent need for the development of less toxic, more selective and targeted therapies for infants with leukemia characterized by translocation of the mixed lineage leukemia (*MLL*) gene. In this study, we performed a cell-based small molecule library screen on an infant MLL-rearranged (MLL-r) cell line, PER-485, in order to identify selective inhibitors for MLL-r leukemia. After screening initial hits for a cytotoxic effect against a panel of 30 cell lines including MLL-r and *MLL* wild-type (MLL-wt) leukemia, solid tumours and control cells, small molecule CCI-007 was identified as a compound that selectively and significantly decreased the viability of a subset of MLL-r and related leukemia cell lines with *CALM-AF10* and *SET-NUP214* translocation. CCI-007 induced a rapid caspase-dependent apoptosis with mitochondrial depolarization within twenty-four hours of treatment. CCI-007 altered the characteristic MLL-r gene expression signature in sensitive cells with downregulation of the expression of *HOXA9*, *MEIS1*, *CMYC* and *BCL2*, important drivers in MLL-r leukemia, within a few hours of treatment. MLL-r leukemia cells that were resistant to the compound were characterised by significantly higher baseline gene expression levels of *MEIS1* and *BCL2* in comparison to CCI-007 sensitive MLL-r leukemia cells.

In conclusion, we have identified CCI-007 as a novel small molecule that displays rapid toxicity towards a subset of MLL-r, CALM-AF10 and SET-NUP214 leukemia cell lines. Our findings suggest an important new avenue in the development of targeted therapies for these deadly diseases and indicate that different therapeutic strategies might be needed for different subtypes of MLL-r leukemia.

## INTRODUCTION

Leukemia characterised by rearrangement of the mixed lineage leukemia (*MLL*) gene is a highly aggressive cancer that occurs in over 70% of infant acute lymphoblastic leukemia (ALL) and 35-50% of infant acute myeloid leukemia (AML) [1, 2]. Notwithstanding the significant progress made in the treatment of childhood leukemia, infant leukemia patients still have a dismal prognosis with a five-year event-free survival of less than 40% [3–5]. Despite the development of infant-specific treatment protocols and incorporation of risk-directed therapy according to prognostic features, the cure rates for infant leukemia have stagnated over recent years [3, 5–7]. The occurrence of a *MLL* gene rearrangement is considered a prognostic factor for high-risk disease, warranting intensified chemotherapy, which often results in problems with toxicity and infection in these high-risk patients [3, 4, 7, 8]. Moreover, the limit for which conventional chemotherapeutics can be intensified to optimize the balance between toxicity and relapse is being reached [7]. It is clear that there is an urgent need for more selective and targeted therapies for *MLL*-rearranged (MLL-r) leukemia, to decrease the side effects of therapy, boost overall survival and improve treatment outcome for these patients.

MLL-r leukemia is characterized by a reciprocal translocation involving the *MLL* gene located on chromosome 11q23 (also known as *KMT2A*, *HRX*, *HTRX1* or *ALL1*), that encodes the MLL1 protein (reviewed in [2, 9]). MLL1 is a histone H3 lysine 4 (H3K4) methyltransferase that associates with several other proteins into a complex responsible for epigenetic regulation of genes through methylation and acetylation of histone core particles [9]. The N-terminal part of the protein is essential for reading and binding the chromatin structure while the C-terminal part performs the enzymatic functions, thereby regulating gene expression and establishing a transcriptional memory system that is essential in normal embryonal development, hematopoiesis, hematopoietic stem cell maintenance, as well as normal cell physiology [10, 11]. Over 79 different fusion partners of MLL1 have been identified and molecularly characterized; the most common fusion partners are AF4 (*AFF1*), AF9 (*MLLT3*), AF10 (*MLLT10*) and ENL (*MLLT1*) corresponding to t(4;11), t(9;11), t(10;11) and t(11;19) translocations respectively and together accounting for almost 80% of all MLL-r leukemias [12]. Infant ALL patients are predominantly characterized by t(4;11), t(11;19), t(9;11), t(10;11), t(6;11) and t(1;11) chromosomal translocations, while for infant AML, t(9;11), t(10;11), t(11;19), t(6;11), t(11;17) and t(1;11) constitute the most common rearrangements [12]. All other *MLL* translocations are relatively rare events. Translocations of the *MLL* gene typically result in the generation of a chimeric protein composed of the N terminal domain of MLL1 and the C terminus of the partner gene protein [1, 2, 9, 10]. This disturbs the normal functioning of the MLL1 protein, causing aberrant histone coding and target gene promoter hyperactivation that in turn result in dysregulated epigenetic and transcriptional programs [9, 13].

In general it is postulated that dysregulated expression of the *HOXA9* gene cluster, which is under tight control by MLL1 during normal hematopoiesis, together with upregulated expression of another *MLL* target gene, the *HOXA9* cofactor *MEIS1*, plays an important role in the development of leukemia as a consequence of the *MLL* translocation [14–18]. Dysregulated *HOXA9* and *MEIS1* expression in hematopoietic progenitor cells has been shown to be leukemogenic [19–24]. Several studies indicate that overexpression of these genes is instrumental in driving the development of MLL-r leukemia and that their suppression is sufficient to compromise MLL-r cancer cell survival [15, 16, 20, 25, 26]. Aberrant expression of the *HOXA* cluster genes has also been reported in MLL-wt leukemias such as leukemias characterized by *CALM-AF10* translocation, *SET-NUP214* fusion and trisomy 8 AML, indicating that deregulation of this pathway might be a common driver in leukemogenesis [27–34]. Indirect evidence for a role of a hyperactivated *HOXA9/MEIS1* pathway in MLL-r leukemia also comes forward from several gene expression studies in patients with MLL-r leukemia [35–39]. These gene expression studies have revealed MLL-r leukemia to be specified by a distinct gene expression signature that is discernible from those of MLL-wt ALL and AML leukemias, regardless of the precise chromosomal translocation and leukemia disease subtypes (ALL and AML) [35–40]. However, several more recent studies in pediatric and infant ALL have shown that, although a fundamental signature is shared by all MLL-r samples, translocation-specific gene expression profiles can be found, as well as the existence of patient subpopulations characterized by specific gene expression profiles, all of which points towards heterogeneity of the disease [41, 42]. Based on these findings it is very well possible that several underlying disease mechanisms and disease-driving pathways are involved to varying degrees in different MLL-r leukemia subtypes (ALL vs AML), for different *MLL* translocations and even breakpoint localizations within the gene [42, 43]. Indeed, throughout the years, other molecules and pathways, besides the *HOXA9/MEIS* pathway, have been postulated to play roles in the survival of MLL-r leukemia cells such as *CMYC*, *BCL2* and the NFκB pathway [43–51].

Recent developments in targeted therapy for MLL-r leukemia have been mainly focused on inhibiting the interaction between MLL1 or the MLL1-fusion protein and collaborating binding partners such as the Disruptor of Telomeric Silencing 1-like (Dot1L) (EPZ4777/EPZ-5676) [52–56], the Multiple Endocrine Neoplasia (Menin) protein (MI-2/MI-3) [57–62] or the WD repeat-containing protein 5 (WDR5) [63–65]. When studied *in vitro*, these inhibitors typically reduce the number of *MLL*-translocated cells over a period of days due to their modes of action through epigenetic modification [53, 54, 59, 63, 64]. A concern with these targets is that they constitute regulators of many physiological processes so that their targeting may result in broad biological effects or toxicity; Dot1L-mediated H3K79 methylation for example is associated with telomere silencing, meiotic checkpoint control and DNA damage response, indicating that the targeting of this protein might pose potential toxicity issues [66–68]. Furthermore, as these targets are also necessary for wild-type MLL1 functioning, and wild-type MLL1 functions are necessary for maintenance of hematopoietic stem cells, it is not unlikely that these compounds could affect these essential cells [43, 69]. In addition, since treatment resistance is a major issue in the clinic and the importance of combination treatment is becoming more evident, the identification of novel drug targets and targetable pathways for MLL-r leukemia will be essential for the design of new, effective therapies for this disease.

In this study we aimed to identify novel small molecule inhibitors of MLL-r leukemia by undertaking an unbiased small molecule library screen against infant MLL-r leukemia cells. By doing so, we identified CCI-007, an inhibitor that selectively targets a subset of MLL-r leukemia cell lines. In addition, this compound affected the viability of CALM-AF10 and SET-NUP214 leukemias. In contrast to the recently developed MLL1-inhibitors for treatment of MLL-r leukemia that typically require days to reduce the viability of MLL-r cells, CCI-007 rapidly suppresses the characteristic *MLL* target gene signature and induces caspase-dependent apoptosis in sensitive cell lines within hours of treatment. These findings suggest an exciting new opportunity for the treatment of these aggressive forms of leukemia.

## RESULTS

### Identification of CCI-007 as a selective inhibitor of MLL-r, CALM-AF10 and SET-NUP214 leukemia

To identify novel compounds that selectively target MLL-r leukemia, a phenotypic screen was performed using a chemical small molecule library composed of 34,000 compounds. The library was screened against an infant MLL-AF4 leukemia cell line (PER-485) in parallel with a human neuroblastoma cell line (BE(2)-C) as representative of a MLL-wt pediatric tumour, using Alamar Blue viability assays at a single dose of 10 μM for each compound. The aim of this parallel screen was to exclude non-MLL-r-selective hits with cytotoxic activity in both cell lines, thereby selecting for compounds more likely to affect the viability of only the MLL-r leukemia cells. 418 compounds were shown to decrease the viability of PER-485 cells while BE(2)-C cell viability was unaffected. Out of these 418 compounds, 245 inhibited PER-485 viability by at least 30%, of which 160 small molecules caused over 70% growth inhibition. Further filtering of these hits was performed by comparing the results of our screen with prior screening data of the same 34,000 small molecule library against solid tumour cell lines 22RV (prostate carcinoma), Mel7 (melanoma) and HeLa (adenocarcinoma) (data not shown). This filtering allowed the exclusion of compounds that reduced the viability of any of these solid tumour cell lines, resulting in the selection of a panel of 30 highly potent small molecules that caused decreased viability of PER-485 cells but not of any of the other MLL-wt, solid tumour cell lines tested.

**Figure 1:**
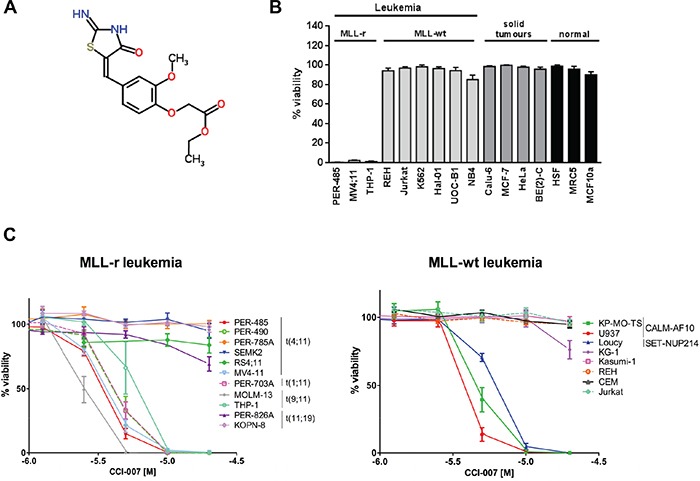
Identification of CCI-007 as a selective inhibitor of MLL-r, CALM-AF10 and SET-NUP214 leukemia **A.** CCI-007 structure **B.** CCI-007 affected the viability of MLL-r cells while normal cells, MLL-wt leukemia and solid tumour cell lines are unaffected. Cytotoxicity of CCI-007 was evaluated in a cell line panel comprising leukemia cell lines with MLL-rearrangements (MLL-r) including MLL-AF4 translocated leukemia (PER-485, MV4;11) and MLL-AF9 translocated leukemia (THP-1), as well as MLL-wt leukemia cell lines (REH, Jurkat, K562, Hal-01, UOC-B1 and NB4), solid tumour cell lines (Calu-6, MCF-7, HeLa and BE(2)-C), non-transformed epithelial (MCF10A) and normal fibroblast cells (HSF and MRC5). The bar graph shows the percentage viability of a panel of cell lines treated with 10 μM CCI-007 as assessed by Alamar Blue assay after 72h in comparison to vehicle treated cells. Each data point represents mean ± SE of at least 3 independent experiments. **C.** CCI-007 reduced the viability of a subset of MLL-r, CALM-AF10 and *SET-NUP214* fusion leukemia cell lines. Cytotoxicity of CCI-007 in a broad concentration range (0.63-20 μM) was evaluated for a panel of MLL-r and MLL-wt leukemia cells (including CALM-AF10 and SET-NUP214 leukemia cells) by Alamar Blue assay after 72h. Viability is calculated compared to vehicle treated cells. Each data point represents mean ± SE of at least 3 independent experiments.

The 30 compounds with selective toxicity for PER-485 cells were then further screened for inhibitory action against a panel of 14 additional cell lines, comprising an additional MLL-AF4, a MLL-AF9 and six MLL-wt leukemia lines, three solid tumour cell lines and three types of normal cells. This screening identified compound CCI-007 as having a selective effect on the viability of the tested MLL-r cell lines, without affecting the viability of the other MLL-wt leukemia cells, solid tumour cell lines and normal cells (Figure 1A, 1B).

More detailed dose-response analysis was performed with CCI-007 on an expanded panel of leukemia cell lines composed of eleven MLL-r (8 ALL, 3 AML) and eight MLL-wt (4 ALL, 4 AML) leukemia cell lines. The MLL-wt leukemia cells included cell lines harbouring a *CALM-AF10* gene fusion (U937 and KP-MO-TS), *SET-NUP214* fusion (Loucy) or chromosome 8 trisomy (KG-1), all previously described to be characterised by *HOXA9* pathway upregulation [27, 28]. These studies showed that the cell lines responsive to CCI-007 were MLL-r (ALL and AML), *CALM-AF10* rearranged (AML) and *SET-NUP214* translocated (ALL) leukemia lines (Figure 1C), Table 1. Within the panel of 11 tested MLL-r cell lines, cell killing with low micromolar IC_50_ values was observed in six cell lines. Sensitivity was independent from the type of MLL translocation or the myeloid/lymphoid nature of the disease: all AML MLL-r cell lines (three out of three, namely MV4;11, MOLM-13 and THP-1 cells) were sensitive, while three out of eight ALL MLL-r cell lines were killed by the compound (PER-485, PER-490 and PER-703A). Similar IC_50_ values were obtained for the two tested CALM-AF10 leukemia cell lines, U937 and KP-MO-TS and the Loucy ALL cell line with *SET-NUP214* translocation. All other tested MLL-wt leukemia cell lines, including the *HOXA9*-overexpressing AML KG-1 cell line (trisomy 8) were insensitive to CCI-007 treatment (Figure 1C), Table 1.

**Table 1:** Differential cytotoxicity of CCI-007 in a panel of human leukemia cell lines

Cell Line			Translocation	Disease	CCI-007 IC_50_ (μM)^a^
**MLL-r**	**ALL**	PER-485	t(4;11)	Infant ALL	**3.5**
		PER-490	t(4;11)	Infant ALL	**4.1**
		PER-703A	t(1;11)	Infant ALL	**4.1**
		PER-785A	t(4;11)	Infant ALL	> 20
		PER-826A	complex t(11;19)	Infant ALL	> 20
		RS4;11	t(4;11)	Pre-B cell ALL	> 20
		SEMK2	t(4;11)	Pre-B cell childhood ALL	> 20
		KOPN-8	t(11;19)	Infant pre-B ALL	> 20
	**AML**	MV4;11	t(4;11)	Childhood AML	**3.7**
		MOLM-13	t(9;11)	AML	**2.5**
		THP-1	t(9;11)	Infant AML	**6.1**
**MLL wt**	**ALL**	Loucy	SET-NUP214	ALL	**6.2**
		CEM		T-cell ALL	> 20
		REH		Pre-B cell ALL	> 20
		Jurkat		Childhood T-cell ALL	> 20
	**AML**	U937	CALM-AF10	AML	**3.8**
		KP-MO-TS	CALM-AF10	AML	**4.7**
		KG-1		AML	> 20
		Kasumi-1	AML-ETO	AML	> 20

a IC_50_ values of CCI-007 against the cell line panel were calculated based on Alamar Blue viability assay data (Figure 1C) by Fit Spline (Point-to-Point) analysis using GraphPad Prism6.

Thus, we have identified a novel compound, CCI-007, that selectively inhibits the viability of a subset of MLL-r, CALM-AF10 and SET-NUP214 leukemia cells, without affecting normal cells, solid tumour lines and other MLL-wt leukemia cells.

### CCI-007 induces a rapid caspase-dependent apoptosis in MLL-r leukemia cell lines

**Figure 2:**
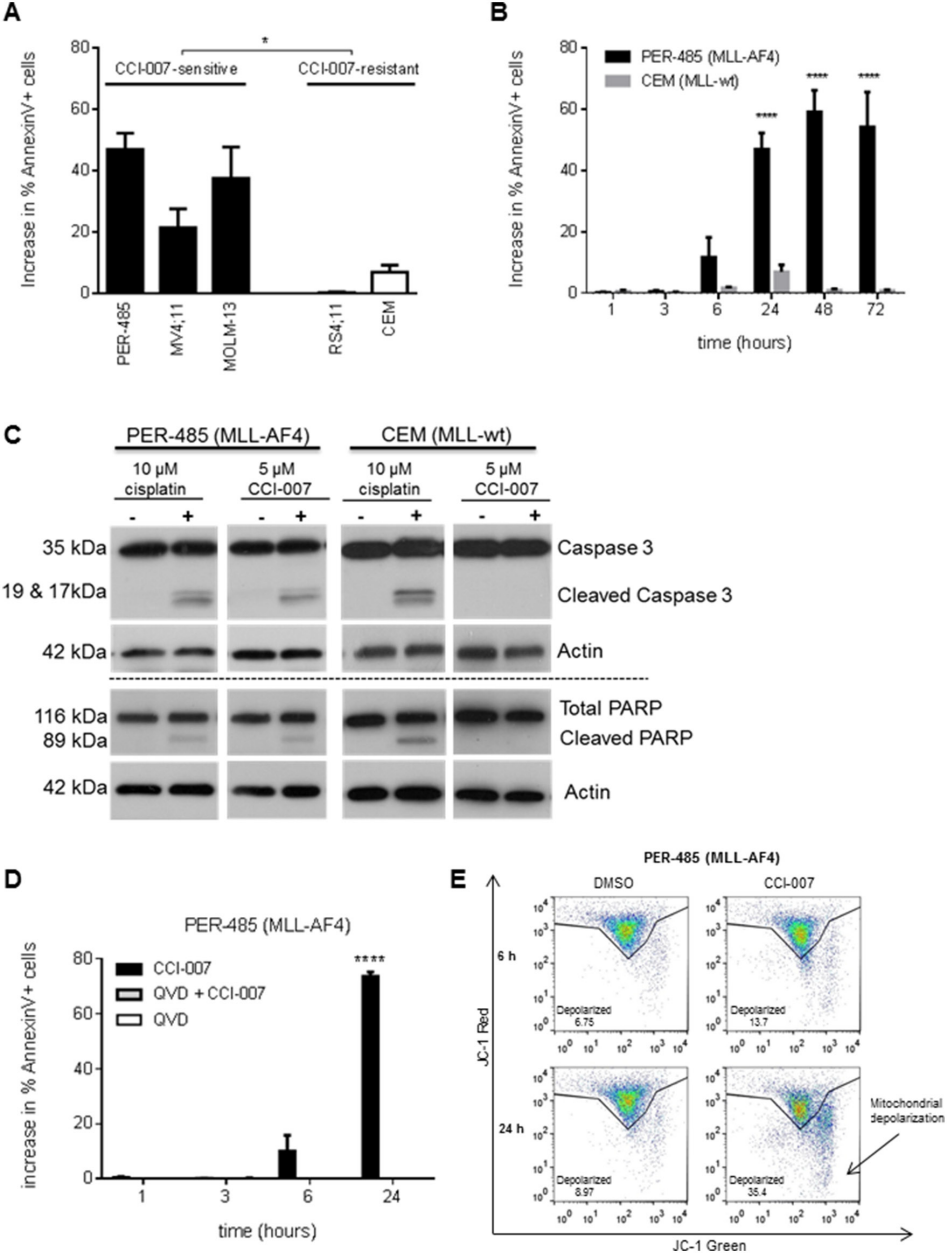
CCI-007 treatment results in caspase-dependent apoptosis in MLL-r leukemia cells **A.** CCI-007 induced an increase in the Annexin V-positive cell population in sensitive MLL-r cell lines PER-485, MV4;11 and MOLM-13, while such an effect was absent in CCI-007 resistant CEM and RS4;11 cells. Bar graph shows mean ± SE of the increase in percentage of Annexin V-positive apoptotic cells as analysed by flow cytometry following treatment of cells with 5 μM CCI-007 compared to vehicle treated cells in at least 3 independent experiments. The mean increase in percentage of Annexin V-positive cells between CCI-007 sensitive and CCI-007 resistant cell line groups was compared by t-test (with Welch correction). *, P<0.05. **B.** CEM and PER-485 cells were treated with 5 μM CCI-007 or DMSO (vehicle) for up to 72h showing that an increase in the percentage of Annexin V-positive cells compared to vehicle treated cells first ensued after 6h CCI-007 treatment in PER-485 cells, reaching a plateau after 48h treatment. The bar graph shows mean ± SE of the increase in percentage of Annexin V-positive apoptotic cells compared to vehicle treated cells, as analysed by flow cytometry in 3 independent experiments. Means were compared between cell lines by t-tests. ****, P<0.0001. **C.** CCI-007 induced PARP cleavage and caspase activation in MLL-r PER-485 cells, but not in MLL-wt CEM cells. A representative immunoblot analysis of total PARP and cleaved PARP, caspase 3 and cleaved caspase 3 protein in PER-485 and CEM cell lines after 24h treatment with 5 μM CCI-007 is shown. Total actin was used as a loading control. Cisplatin was included as a positive control for caspase-dependent apoptosis induction. Western blotting was performed at least 2 times in independent experiments. **D.** Induction of apoptosis by CCI-007 in MLL-r cells is dependent on caspase activity. PER-485 were pre-treated with 10 μM pan-caspase inhibitor Q-VD-OPh for 2h prior to treatment with 5 μM CCI-007 for 1, 3, 6 and 24h. Bar graph depicts mean ± SE of the increase in percentage of Annexin V-positive PER-485 cells following treatment with CCI-007 and Q-VD-OPh either alone or in combination, at various time points as measured by flow cytometry in 3 independent experiments. Mean percentages of Annexin V-positive cells between treatment groups were compared by ANOVA. ****, P<0.0001. **E.** CCI-007 induces mitochondrial depolarization in sensitive MLL-r cells within 24h of treatment. Representative JC-1 staining results of DMSO or 5 μM CCI-007 treated PER-485 cells after 6h or 24h treatment as determined by flow cytometry. An increase in depolarization as evidenced by a shift in JC-1 red to green signal is observed in PER-485 cells within 24h. Representative data from 3 independent experiments are shown.

To elucidate the mechanism by which CCI-007 decreases cell viability of MLL-r leukemia cells, the impact of CCI-007 on apoptosis and cell cycle progression was investigated. A significant increase in the population of Annexin V-positive cells was detected in MLL-r cell lines sensitive to CCI-007 (PER-485, MOLM-13, MV4;11) upon CCI-007 treatment for 24h, while such an effect was not observed after treatment of CCI-007 resistant leukemia cell lines (CEM and RS4;11) (Figure 2A). The population of Annexin V-positive cells became apparent in PER-485 cells as early as 6h post treatment with the small molecule, with around half of the cells undergoing apoptosis by 24h post treatment (Figure 2B). To determine whether this increase in Annexin V-positive cells was associated with caspase-dependent apoptosis, PER-485 and CEM cells were incubated with CCI-007 and the presence of cleaved caspase 3 and cleaved PARP as markers for apoptosis was examined. CCI-007 induced caspase 3 and PARP cleavage in PER-485 cells to a degree similar to that induced by cisplatin treatment, while no apoptotic response to CCI-007 was observed in CEM cells (Figure 2C). To further confirm the occurrence of caspase-mediated apoptosis, PER-485 cells were pre-incubated with the pan-caspase inhibitor Q-VD-OPh. Inhibition of caspase activation completely abrogated the increase in Annexin V-positive PER-485 cells suggesting that CCI-007 induces a caspase-dependent apoptosis (Figure 2D). To determine whether CCI-007 treatment also affected mitochondrial membrane potential, treated PER-485 cells were stained with JC-1 followed by flow cytometry analysis. Following CCI-007 treatment for 24h, significant mitochondrial depolarization was observed in PER-485 cells as evidenced by a shift in JC-1 signal (Figure 2E).

Analysis of cell cycle progression upon CCI-007 treatment of PER-485 cells showed no biologically relevant changes in the percentages of cells in different phases of cell cycle up to 72h after treatment (Supplementary Figure S1). After 24h treatment, a very small, but significant increase in the percentage of cells in G_0_/G_1_-phase and concomitant decrease in percentage of cells in S-phase was demonstrated. The absence of cell cycle changes at later time points after treatment indicates that the main effect of CCI-007 is apoptosis induction.

Taken together, these data show that CCI-007 rapidly induces caspase-dependent apoptosis in sensitive MLL-r leukemia cell lines within 24h of treatment.

### CCI-007 downregulates expression of MLL-r leukemia associated survival genes

**Figure 3:**
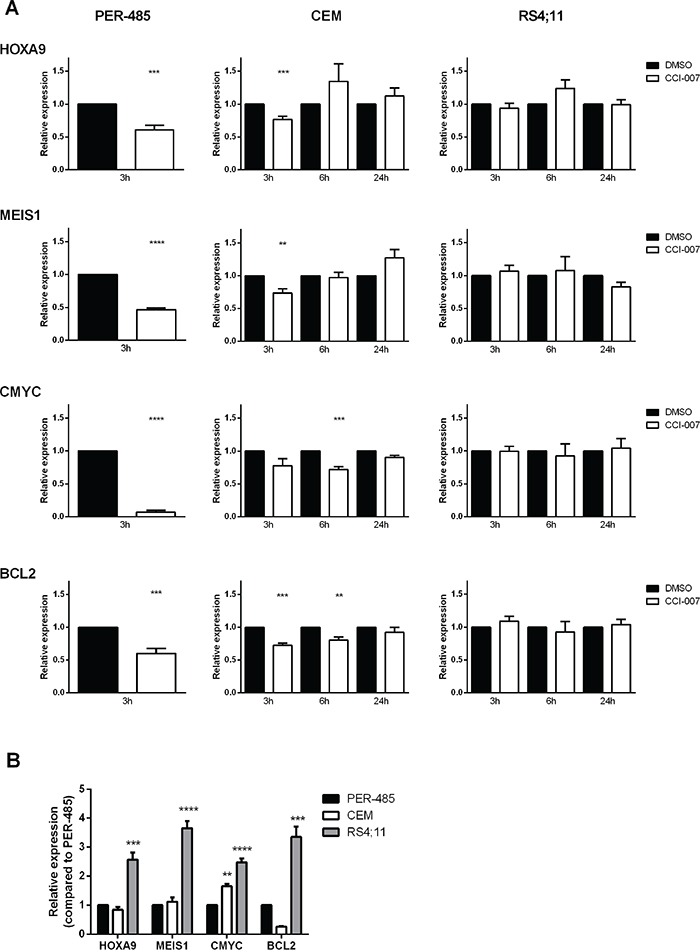
CCI-007 decreases mRNA expression of MLL target genes in sensitive MLL-r leukemia cells **A.** CCI-007 sensitive MLL-r PER-485, CCI-007 resistant MLL-wt CEM and CCI-007 resistant MLL-r RS4;11 cells were treated with 5 μM CCI-007 for 3h. *HOXA9*, *MEIS1*, *CMYC* and *BCL2* mRNA levels were assayed by quantitative real-time RT-PCR and relative expression was calculated using the ΔΔCt method. Gene expression was normalized against housekeeping genes and expressed relative to DMSO vehicle control. Assays were run in triplicate. Each data point represents the mean ± SE of at least 3 independent experiments. Mean relative expressions between treatment groups were compared by t-tests. As after 6h CCI-007 treatment, the PER-485 cells were already undergoing apoptosis as evidenced by increases in Annexin V-positive cell population, the levels of housekeeping genes were affected. This prevented accurate gene expression level determination in those cells for time points later than 3h. **B.** PER-485, CEM and RS4;11 cells were harvested when in exponential growth phase to assess baseline gene expression levels. *HOXA9*, *MEIS1*, *CMYC* and *BCL2* mRNA levels were assayed by quantitative real-time RT-PCR and relative expression was calculated using the ΔΔCt method. Gene expression was normalized against housekeeping genes and expressed relative to PER-485 cells. Assays were run in triplicate. Each data point represents the mean ± SE of at least 3 independent experiments. Mean baseline expression levels between cell lines were compared by ANOVA followed by multiple comparisons. Asterisks represent P-values of comparisons between baseline gene expression levels in the corresponding cell line and PER-485 cells. *, P<0.05; **, P<0.01; ***, P<0.001; ****, P<0.0001.

MLL-r leukemia is characterised by deregulated gene expression that reportedly entails increased expression levels of *HOXA9*, *MEIS1, CMYC* and *BCL2*, which have been suggested to play important roles in MLL-r leukemia cell survival [44–46]. To assess whether CCI-007 affects expression levels of these important MLL-r leukemia survival genes, quantitative real-time RT-PCR was used with RNA extracted from cells treated with the compound. CCI-007 was shown to reduce *HOXA9*, *MEIS1, CMYC* and *BCL2* mRNA levels within a few hours after treatment in sensitive MLL-r (PER-485, MOLM-13, MV4;11) and CALM-AF10 leukemia (KP-MO-TS) cell lines, while the effects of CCI-007 treatment on expression levels of those genes in resistant leukemia cells were either minimal or absent (Figure 3A and Supplementary Figure S2). The CCI-007 resistant MLL-r RS4;11 cells, which were shown to express higher baseline levels of all four genes compared to PER-485 cells (Figure 3B), did not exhibit significant changes in any of the MLL target gene mRNAs upon treatment with CCI-007. For the CCI-007 resistant MLL-wt CEM cells, changes in mRNA levels were evident after CCI-007 treatment but these were less pronounced compared to PER-485 cells (Figure 3A). Moreover, for the later time points after CCI-007 treatment at which apoptosis was clearly occurring in the CCI-007 sensitive PER-485 cells (6h and 24h), the level of the MLL target gene mRNAs returned to normal in CEM cells, indicating that, in contrast to the sensitive MLL-r cells, the initial decreases in mRNA amounts were only transient and CEM cells were able to recover (Figure 3A). To further investigate the cellular response to CCI-007, PER-485 cells were exposed to short term treatment (6h) with CCI-007 after which the compound was removed and cells were allowed to recover. The expression levels of *HOXA9*, *MEIS1*, *CMYC* and *BCL2* genes were rapidly restored after compound removal (Supplementary Figure S3). The normalisation of the MLL target gene expression occurred in parallel to recovery of cell viability and proliferation, providing further support for the instrumental role of these genes in MLL-r cell survival and proliferation (Supplementary Figure S3).

**Figure 4:**
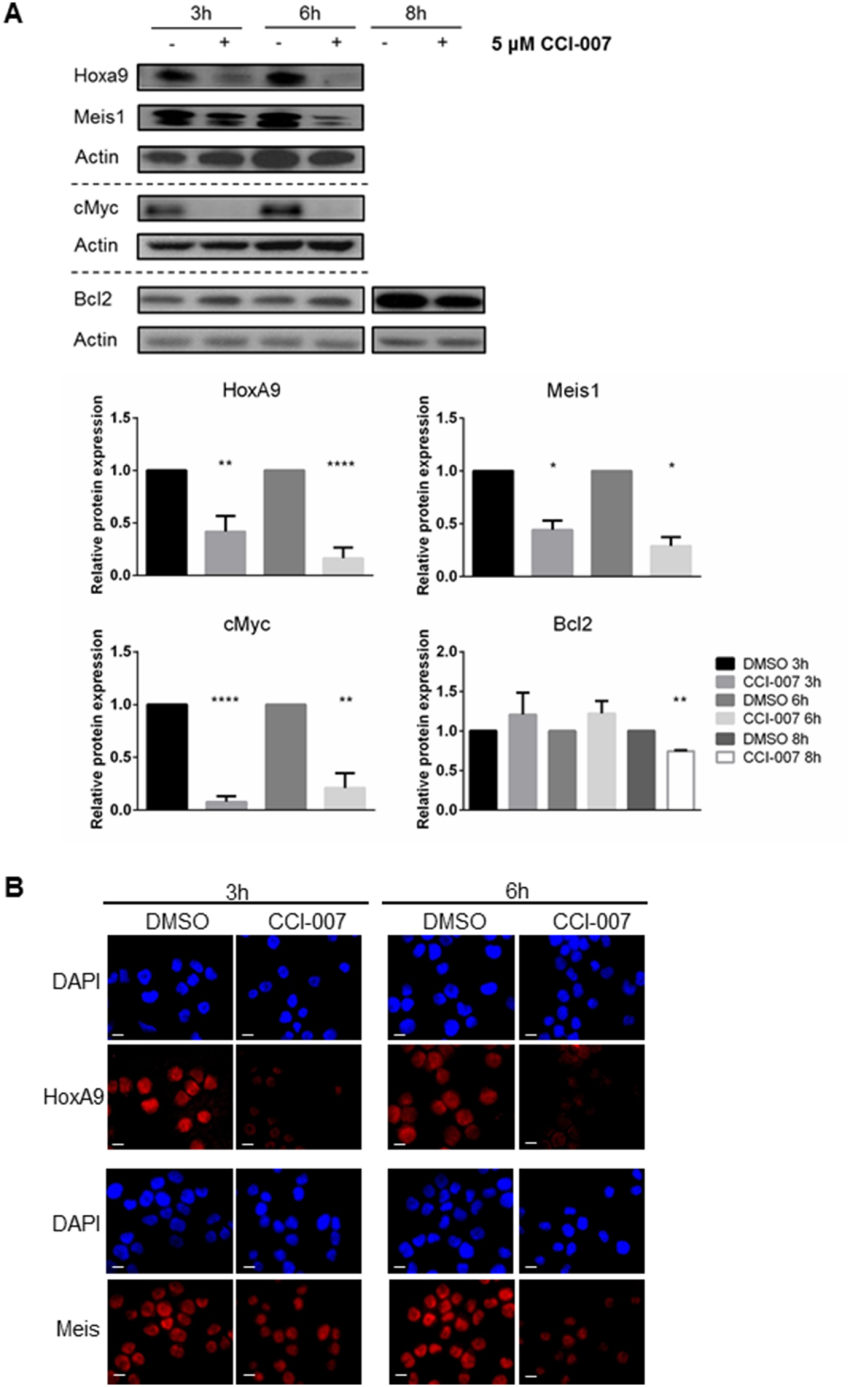
CCI-007 decreases expression of MLL-r downstream leukemogenic proteins **A.** Representative immunoblot showing decreased protein expression of HoxA9, Meis1, cMyc and Bcl2 in PER-485 treated with 5 μM CCI-007 for 3h, 6h and 8h. Total actin was used as a loading control. At least 2 (Meis1, Bcl2) or 3 (HoxA9, cMyc) independent experiments were performed. Semi-quantitative densitometry analysis was performed on all replicates. Histograms display the mean protein expression levels relative to the DMSO control treatment for each time point (normalized to actin) ± SEM. *, P<0.05; **, P<0.01; ****, P<0.0001. **B.** Immunofluorescent staining for HoxA9 and Meis on sensitive PER-485 cells after a 3h and 6h treatment with 5 μM CCI-007 or DMSO as vehicle control. DAPI (blue) stains nuclei. Red signal corresponds to HoxA9 or Meis. Scale bar represents 10 μm.

Western blotting and immunofluorescence subsequently confirmed that the observed decreases in MLL target gene mRNA levels in PER-485 cells functionally resulted in decreased cellular levels of these proteins (Figure 4A, 4B). The protein levels of HoxA9, Meis1 and cMyc began to decrease by 3h post treatment, while a decline in Bcl2 protein levels was observed after 8h treatment (Figure 4A, 4B).

The rapidity with which the decrease in MLL target gene expression in PER-485 cells was observed (within a few hours after treatment), and the fact that the decrease occurred before Annexin V upregulation (Figure 2B), suggests that the CCI-007-induced altered gene expression is independent of and not secondary to induction of apoptosis. To further confirm this, cells were incubated with CCI-007 in the presence and absence of pan-caspase inhibitor Q-VD-OPh. As expected, inhibition of caspase-dependent apoptosis did not prevent the CCI-007-induced decrease in mRNA levels of *HOXA9*, *MEIS1*, *CMYC* and *BCL2,* confirming that changes in gene expression occurred before apoptosis (Supplementary Figure S4).

### CCI-007 reverses the MLL-r and CALM-AF10 leukemia target gene signatures

**Figure 5:**
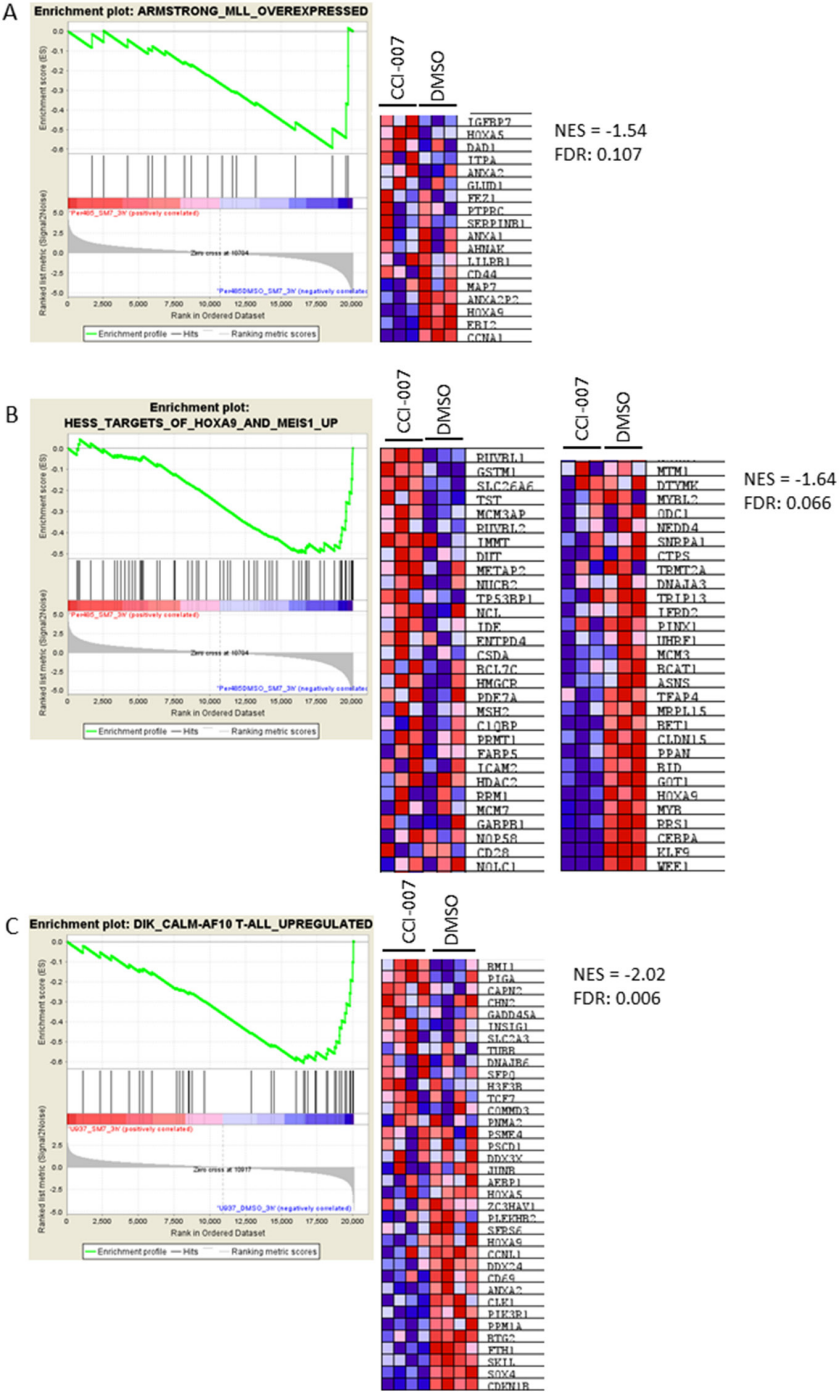
CCI-007 reverses the MLL-r and CALM-AF10 leukemia gene signatures Gene Set Enrichment Analysis (GSEA) analysis was performed on the lists of differentially expressed genes of CCI-007 treated PER-485 (A, B) and U937 (C) cells compared to vehicle treated cells. **A.** CCI-007 treatment of PER-485 cells is associated with a downregulation of genes that are upregulated in human MLL-r leukemias (compared to MLL-wt ALL leukemias) (Armstrong *et al.,* Nat Genetics 2002). **B.** CCI-007 treatment of PER-485 cells is associated with a downregulation of genes described as *HOXA9* and *MEIS1* target genes (Hess *et al.,* Blood 2006). **C.** Treatment of U937 cells resulted in downregulation of genes defined in the characteristic CALM-AF10 gene signature described by Dik *et al.*, Leukemia 2005. The heat maps show genes comprising the leading edge of the GSEA plot. Red indicates high expression, blue indicates low expression. A False Discovery Rate (FDR) below 0.25 is considered significant. NES, Normalized Enrichment Score.

To further confirm that CCI-007 reverses the characteristic MLL-r leukemia gene expression signature, RNA was isolated from PER-485 cells treated with CCI-007 for 3h and microarray-based whole genome expression profiling was performed. Gene Set Enrichment Analysis was used to compare genes downregulated following 3h CCI-007 treatment of PER-485 cells with genes overexpressed in MLL-r human leukemias (compared to MLL-wt ALL leukemias) [35]. This analysis showed that genes downregulated by CCI-007 in PER-485 cells demonstrated strong enrichment for genes upregulated in MLL-r leukemia (NES = −1.54; FDR:0.107, Figure 5A) as well as for *HOXA9/MEIS1* target genes (NES = −1.64; FDR:0.066) (Figure 5B) [70]. A similar analysis on the sensitive CALM-AF10 leukemia cells, U937, treated with CCI-007 for 3h indicated that CCI-007 treatment reversed the characteristic CALM-AF10 leukemia gene signature (NES = −2.02; FDR: 0.006), thus affecting the characteristic gene expression profile of the disease (Figure 5C) [71]. These observations further confirm that CCI-007 effectively reverses the MLL-r and CALM-AF10 leukemia gene signatures and thereby reduces the expression levels of leukemogenic genes important for these diseases.

### CCI-007 sensitive MLL-r cell lines have lower baseline *MEIS1* and *BCL2* expression levels than CCI-007 resistant MLL-r cell lines

**Figure 6:**
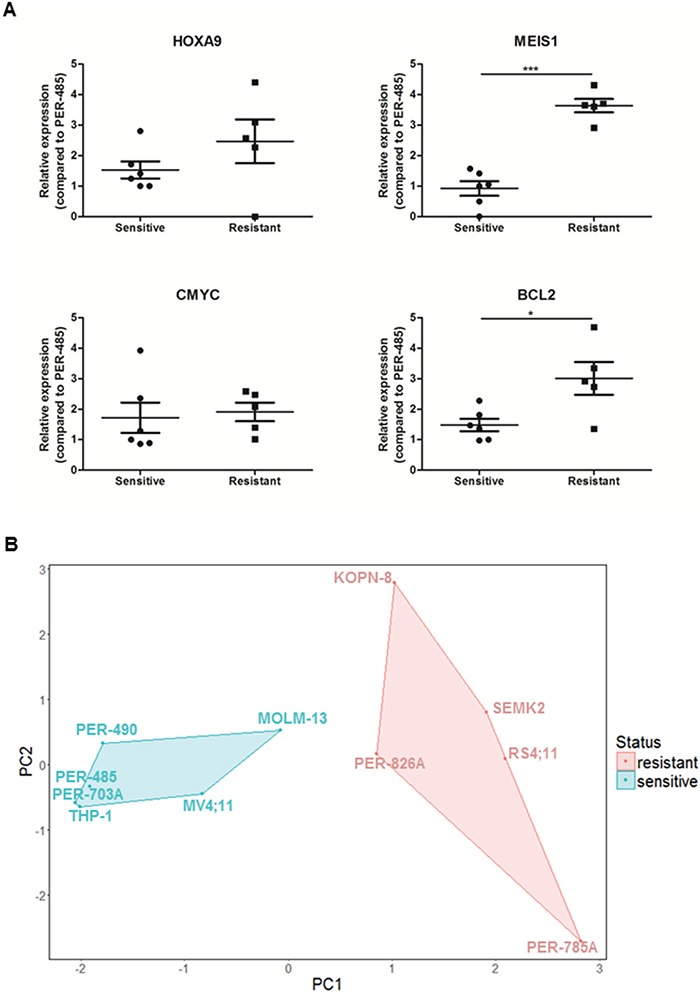
CCI-007 sensitive MLL-r leukemia cells have lower baseline MEIS1 and BCL2 expression levels than resistant MLL-r leukemia cells Leukemia cells were harvested in the exponential growth phase to assess baseline expression levels of *HOXA9*, *MEIS1*, *CMYC* and *BCL2*. CCI-007 sensitive MLL-r leukemia cells (n=6) constitute PER-485, PER-490, PER-703A, MV4;11, MOLM-13 and THP-1, while PER-785A, RS4;11, SEMK2, PER-826A and KOPN-8 are CCI-007 resistant MLL-r cell lines (n=5). *HOXA9*, *MEIS1*, *CMYC* and *BCL2* mRNA levels were assayed by quantitative real-time RT-PCR and relative expression was calculated using the ΔΔCt method. Gene expression was normalized against housekeeping genes and expressed relative to PER-485 baseline expression levels. Assays were run in triplicate. **A.** Each data point represents the mean ± SE of at least 3 independent experiments. Means were compared between groups by t-tests. *, P<0.05; ***, P<0.001. **B.** PCA clustered MLL-r cell lines into two distinct clusters, corresponding to CCI-007 sensitive and resistant cell lines.

In order to identify the basis of selectivity of CCI-007 towards a subgroup of MLL-r leukemia cell lines, baseline expression levels of important leukemia associated genes *HOXA9*, *MEIS1*, *CMYC* and *BCL2* were determined by quantitative real-time RT-PCR and relative baseline expression levels were compared between CCI-007 sensitive and resistant cell lines. Interestingly, this analysis revealed that, within the MLL-r leukemia cell lines, CCI-007 resistant MLL-r leukemia cells had significantly higher baseline expression levels of *MEIS1* and *BCL2* compared to sensitive cell lines, indicating selective targeting of MLL-r cells with lower baseline *MEIS1* and *BCL2* expression levels by CCI-007 (Figure 6A). Similarly, a trend for higher mRNA levels in resistant MLL-r leukemia cell lines was noted for *HOXA9*, albeit not significant. After performing a Principal Component Analysis (PCA) on the gene expression data for *HOXA9*, *MEIS1*, *CMYC* and *BCL2* for all MLL-r cell lines, the MLL-r cell lines segregated into two well defined and separated clusters, one containing the CCI-007 sensitive MLL-r cells and the other consisting of the CCI-007 resistant cell lines (Figure 6B). The percentages of contribution of the individual gene expression data to Principal Component 1 (PC1) were 4%, 64%, 6% and 26% for *HOXA9*, *MEIS1*, *CMYC* and *BCL2* respectively, while for Principal Component 2 (PC2), the contribution percentages were 76%, 1%, 2% and 21% for *HOXA9*, *MEIS1*, *CMYC* and *BCL2*, respectively. Overall, *MEIS1* had the greatest combined contribution followed by *HOXA9*, *BCL2* and *CMYC* (in decreasing contribution). Baseline expression levels of *MEIS1*, *BCL2* and *HOXA9* are thus major contributors to the segregation of the MLL-r cell lines based on CCI-007 sensitivity, while *CMYC* baseline expression levels do not seem to play a major role. These data suggest that CCI-007 targets a subpopulation of MLL-r leukemia cell lines with a specific baseline gene expression profile of *HOXA9*, *MEIS1* and *BCL2* MLL target genes.

When comparing gene expression levels of *HOXA9*, *MEIS1*, *CMYC* and *BCL2* across the broad leukemia cell line panel, no significant differences could be detected in baseline expression levels of the four genes between leukemia cell lines sensitive and resistant to CCI-007 (Supplementary Figure S5A). In addition, PCA on the baseline gene expression data did not segregate the cells into separate clusters in terms of response to CCI-007 (Supplementary Figure S5B).

### Resistance to CCI-007 can occur by upregulation of MLL target gene expression

**Figure 7:**
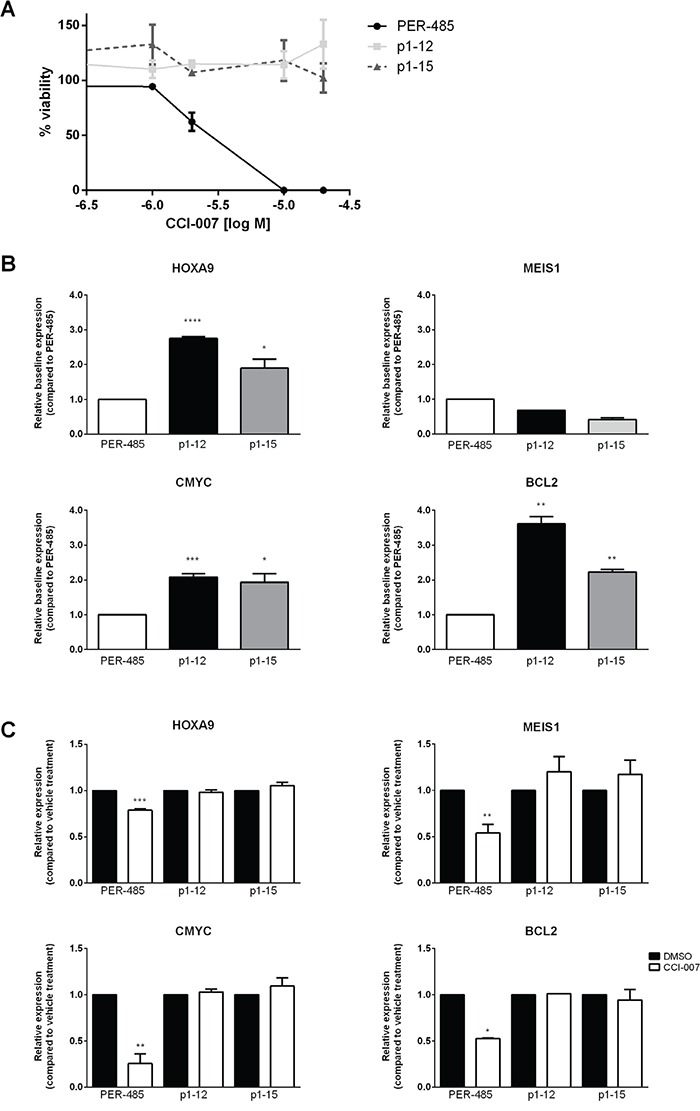
CCI-007 resistant cell lines display upregulated HOXA9, CMYC and BCL2 expression **A.** Dose response of CCI-007 sensitive PER-485 and CCI-007 resistant p1-12 and p1-15 cells to CCI-007 treatment as assessed in Alamar Blue assay after 72h in comparison to vehicle treated cells. Each data point represents mean ± SE of at least 3 independent experiments. **B.** CCI-007 resistant cell lines p1-12 and p1-15 have increased basal *HOXA9*, *CMYC and BCL2* mRNA levels compared to the parental CCI-007 sensitive PER-485 cell line. *HOXA9*, *MEIS1, BCL2* and *CMYC* mRNA levels were assayed by quantitative real-time RT-PCR and relative expressions were calculated using the ΔΔCt method. Gene expressions were normalized against housekeeping genes and expressed relative to parental PER-485 cells. Assays were run in triplicate. Each data point represents the mean ± SE of at least 2 independent experiments. T-tests were used to compare relative baseline expression in the resistant lines with relative expression in the parental PER-485 cell line. *, P<0.05; **, P<0.01; ***, P<0.001; ****, P<0.0001. **C.** CCI-007 resistant cell lines p1-12 and p1-15 do not lower *HOXA9*, *MEIS1*, *CMYC or BCL2* mRNA levels upon treatment with CCI-007 compared to vehicle treated cells. p1-12 and p1-15 were treated with 5 μM CCI-007 or vehicle for 3h in parallel with the parental PER-485 cell line. *HOXA9*, *MEIS1, BCL2* and *CMYC* mRNA levels were assayed by quantitative real-time RT-PCR and relative expressions were calculated using the ΔΔCt method. Gene expressions were normalized against housekeeping genes and expressed relative to vehicle treated cells. Assays were run in triplicate. Each data point represents the mean ± SE of at least 2 independent experiments. T-tests were used to compare relative expression between CCI-007 and vehicle treated cells. *, P<0.05; **, P<0.01; ***, P<0.001.

To obtain further insight into the mode of action of and response to CCI-007, CCI-007 resistant cell lines were generated by culturing PER-485 cells in high concentrations of the compound (IC_90_). Resistance to CCI-007 was not readily achieved and multiple (>12) selection rounds, including additional rounds at incrementally increased doses, were needed to generate two resistant pools of PER-485 cells, namely p1-12 and p1-15, that demonstrated complete resistance at doses up to 40 μM CCI-007 (Figure 7A). Quantitative real-time RT-PCR for MLL target genes indicated that each of the CCI-007-resistant PER-485 pools presented with significantly increased baseline expression levels of *HOXA9*, *CMYC and BCL2* mRNA compared to the parental PER-485 cells (Figure 7B). No significant changes were seen for the mRNA levels of *MEIS1* in the resistant pools (Figure 7B). Moreover, treatment of the p1-12 and p1-15 cells with CCI-007 did not induce the decreases in MLL target gene mRNA levels that were observed for the parental PER-485 cells (Figure 7C).

Thus, by continuous culturing in CCI-007, the selected resistant cell lines have adopted mechanisms leading to high baseline expression levels of *HOXA9*, *CMYC* and *BCL2* that are not affected by treatment with CCI-007, reminiscent of the effect seen in the naturally resistant MLL-r cells. These results provide additional support for the critical role of *HOXA9*, *CMYC* and *BCL2* for MLL-r leukemia cell survival and the potential of CCI-007 to affect the expression of those genes in a subset of MLL-r cells to induce cell killing.

## DISCUSSION

MLL-r leukaemia is a highly aggressive disease with poor prognosis for which novel targeted therapies are urgently needed. In this study, we report the discovery of a molecule, CCI-007, that markedly affects the viability of a subset of MLL-r leukemia cells, as well as CALM-AF10 and SET-NUP214 leukemia cells. The rapid response to CCI-007 treatment in the sensitive MLL-r cell lines, involving a marked reduction in *HOXA9*, *MEIS1, CMYC and BCL2* mRNA levels within the first hours after treatment, a concomitant decrease in protein levels and induction of caspase-dependent apoptosis within 24 hours after treatment, is in sharp contrast to the effects of newly developed MLL1-complex inhibitors that target proteins that are recruited by the aberrant MLL1 fusion proteins or germline MLL1 protein such as Dot1L, Menin or WDR5. While each of these inhibitors has been described to decrease mRNA levels of *HOXA9*, *MEIS1* or other MLL target genes, the reduction in expression of these genes requires several days of treatment to become apparent [53, 54, 59, 61–65]. Moreover, in contrast to the rapid induction of caspase-dependent apoptosis with minimal effects on cell cycle progression seen following CCI-007 treatment, the primary effects of these MLL1-complex inhibitors include induction of cell cycle block and an increase in differentiation which typically occur over the time course of several days [53, 54, 59, 61–65]. The mechanism of action of CCI-007 as well as its selectivity towards a subpopulation of MLL-r leukemia cells is thus clearly distinct from that of any of these recently developed MLL1-inhibitors.

The long existing dogma within MLL-r leukemia research that *HOXA9* is consistently highly expressed in all MLL-r leukemias has been challenged by several recent studies indicating that *HOXA9 per se* might not be required for all MLL-rearranged pediatric and infant ALL leukemias [41, 42]. It is conceivable that while a panel of genes contributes to the survival of MLL-r leukemia, the individual contributions of those genes to disease development might depend on the chromosomal translocation type or even breakpoint localizations [10, 42]. This is supported by the discovery of translocation specific gene expression profiles associated with different types of MLL translocation in infant MLL-r ALL which indicates that apart from the background gene expression pattern driven by *MLL* gene, the fusion partner determines additional changes in gene expression characteristic for the type of MLL translocation [41]. In general, several other genes, such as *BCL2* have previously been described to be important for MLL-r tumour cell development or survival in such a way that suppression of gene expression is able to abolish MLL-r leukemia by proliferation arrest and/or apoptosis induction [45–47]. These recent insights highlight a heterogeneity of *MLL*-rearranged leukemia and imply that different disease subtypes may require specific pharmacological intervention targeting the corresponding underlying pathogenetic mechanisms [10]. In view of this postulated heterogeneity of MLL-r leukaemia, it is interesting to note the particular selectivity of CCI-007 against a subset of MLL-r leukemia cell lines as well as CALM-AF10 and SET-NUP214 leukemia. As CALM-AF10 and SET-NUP214 leukemias are characterized by *HOXA9*-overexpression and dependency on the *HOXA9* pathway, we tested for differences in baseline expressions of *HOXA9* and *MEIS1* between CCI-007 sensitive and resistant leukemia cells, but could not detect any significant differences indicating that the compound does not simply target *HOXA9/MEIS1* overexpressing leukemia cells as such. However, when comparing baseline expression levels of these genes within the MLL-r leukemia cell line group itself, it became evident that the MLL-r cell lines that are sensitive to CCI-007 have significantly lower baseline levels of *MEIS1* and *BCL2* mRNA and cluster separately from resistant cell lines based on expression levels of *HOXA9*, *MEIS1* and *BCL2*, indicating that this compound targets a particular subpopulation of MLL-r leukemia cells.

These findings indicate that resistance to CCI-007 within the broad leukemia panel is brought about by different mechanisms for MLL-r and MLL-wt leukemia cells and indeed, the expression levels of *HOXA9*, *MEIS1*, *CMYC* and *BCL2* responded differently upon CCI-007 treatment of sensitive MLL-r versus resistant MLL-r and resistant wt-MLL leukemia cells. Firstly, in the MLL-wt CEM cells, CCI-007 treatment induced an initial decrease in expression levels of *HOXA9*, *MEIS1*, *CMYC* and *BCL2*. However, these decreases were transient, the cells did not undergo apoptosis in response to these temporarily lowered gene expression levels and the gene expression normalized again within hours of exposure, despite continued presence of the compound. These data indicate that the survival of MLL-wt leukemia cells is not dependent upon expression of these genes and that the cells are dependent on other drivers for survival or that these cells have fast-acting rescue mechanisms to recover.

Secondly, a different resistance mechanism seems to be in place within the group of MLL-r leukemia cells. Both the naturally resistant MLL-r cell lines, as well as the selected PER-485 resistant cell lines p1-12 and p1-15, are characterised by very high baseline expression levels of one or more of the pro-survival *HOXA9*, *MEIS*, *CMYC* and *BCL2* genes and treatment with the compound does not affect these high expression levels at any point in time. These findings point towards the MLL-r resistant cells possessing signaling pathways or rapidly engaged rescue mechanisms that result in maintained expression levels of these genes despite CCI-007 treatment.

Based on our dataset, the selectivity of the compound within MLL-r leukemia cell lines is not brought about by the presence of certain chromosomal translocations or breakpoints. CCI-007 sensitive MLL-r cell lines constitute both ALL as well as AML cells, and represent diverse MLL translocation partners, as the panel of sensitive cell lines was characterized by the presence of t(4;11), t(9;11) and t(1;11) translocations. Whilst the precise mechanism by which CCI-007 induces a rapid decrease in mRNA expression levels of MLL target genes is currently unknown, the short time frame for mRNA decreases induced by CCI-007 suggests that the compound is unlikely to work through an effect on the aberrant histone methylation activity of MLL1 fusion proteins. Instead, CCI-007 may act at the level of the aberrant transcriptional activity of the MLL1 fusion protein, as it has been shown that fusion of MLL1 with the most common MLL fusion partners (AF4, AF9, ENL and ELL) results in an increase in the transcriptional elongation activity, thereby bypassing the normal initiation-to-elongation checkpoints [72, 73] [74] [10]. If CCI-007 affects this MLL-r associated increased transcriptional elongation activity, this could explain the rapid decrease in mRNA levels of MLL target genes and the subsequent drop in protein levels of these important survival proteins.

Given the unique mechanism of action of CCI-007 by comparison with the recently developed MLL-complex inhibitors, it will be important to test its activity *in vivo*. CCI-007 itself lacks sufficient metabolic stability to be suitable for *in vivo* use, hence more stable and potent compounds based on CCI-007 are currently under development. Preliminary focused library screening showed that several molecules with similar structure to CCI-007 have identical effects on the viability of CCI-007 sensitive MLL-r and CALM-AF10 cell lines, indicating the importance of a specific core structure for anti-MLL-r activity. Further studies will delineate which molecular groups are important for activity, guiding the way towards development of a CCI-007 analogue with better drug-like properties. Future efforts will also be focused on the identification of the specific target and pathway engaged by CCI-007 in order to identify other therapeutic targets or inhibitors with potential clinical application as well as to discover markers that can be used to identify patients that would be responsive to treatment with the compound.

In conclusion, we have identified a novel molecule that rapidly induces caspase-dependent apoptosis in a subpopulation of MLL-r, CALM-AF10 and SET-NUP214 leukemias. To the best of our knowledge, this is the first selective MLL-r leukemia inhibitor with such a short time frame of action and particular selectivity. Identification of the underlying mode of action of the compound will allow the identification of a MLL-selective, targetable pathway that could have important clinical impact for patients who suffer from this aggressive disease.

## MATERIALS AND METHODS

### Human cell lines

A panel of 30 human cell lines including MLL-r leukemias, MLL-wt leukemias, solid tumour cell lines and control, non-malignant cells was used for *in vitro* experiments (Table 2). The PER-485, PER-490, PER-703A, PER-785A, PER-826A cell lines were established from infant patients with pre-B cell acute lymphoblastic leukemia [75, 76]. The MOLM-13 cell line was kindly provided by R. D'Andrea, IMVS, South Australia, Australia. THP-1 cells were a kind gift from W. Jessup, Centre for Vascular Research, New South Wales, Australia. Hal-01 and UOC-B1 cells were kindly provided by A. Thomas, Dana-Farber Cancer Institute, Massachusetts, USA. BE(2)-C cells were a kind gift from J. Biedler, Memorial Sloan-Kettering Cancer Centre, NY, USA. All other cells were purchased from ATCC (Manassas, Virginia, USA) or DSMZ (Braunschweig, Germany). Cells were maintained in DMEM, IMDM or RPMI 1640 media containing 20% or 10% Foetal Calf Serum (FCS), 1% Non-essential Amino Acid Mix (100x) and 1% 100mM Sodium Pyruvate Solution (100x) (Life Technologies, Scoresby, Victoria, Australia).

**Table 2:** Panel of cell lines used for *in vitro* experiments

Cell Line	MLL status	Disease	Supplier	Authentication^a^
PER-485	t(4;11)	Infant ALL	Kees UR et al. Mol Cancer Ther 2003	STR profiling 22/03/2016
PER-490	t(4;11)	Infant ALL	Kees UR et al. Mol Cancer Ther 2003	STR profiling 22/03/2016
PER-703A	t(1;11)	Infant ALL	Kees UR et al. Mol Cancer Ther 2003	STR profiling 22/03/2016
PER-785A	t(4;11)	Infant ALL	Kees UR et al. Mol Cancer Ther 2003	STR profiling 22/03/2016
PER-826A	complex t(11;19)	Infant ALL	Kees UR et al. Mol Cancer Ther 2003	STR profiling 22/03/2016
MV4;11	t(4;11)	Childhood AML	ATCC	STR profiling 05/07/2013
RS4;11	t(4;11)	Pre-B cell ALL	ATCC	STR profiling 22/03/2016
SEMK2	t(4;11)	Pre-B cell childhood ALL	ATCC	STR profiling 05/07/2013
MOLM-13	t(9;11)	AML	R D'Andrea, IMVS, South Australia, Australia	STR profiling 15/08/2015
THP-1	t(9;11)	Infant AML	W Jessup, Centre for Vascular Research, NSW, Australia	STR profiling 22/03/2016
KOPN-8	t(11;19)	Infant pre-B ALL	DSMZ	STR profiling 22/03/2016
U937	MLL-wt CALM-AF10	AML derived from histiocytic lymphoma	ATCC	STR profiling 17/06/2014
KP-MO-TS	MLL-wt CALM-AF10	AML	Imamura T [77]	NA^b^
Loucy	MLL-wt SET-NUP214	T-cell ALL	ATCC	STR profiling 22/03/2016
KG-1	MLL-wt	AML	ATCC	STR profiling 22/03/2016
Kasumi-1	MLL-wt	AML	ATCC	STR profiling 22/03/2016
REH	MLL-wt	Pre-B cell ALL	ATCC	STR profiling 22/03/2016
CEM	MLL-wt	T-cell ALL	ATCC	STR profiling 09/05/2013
Jurkat	MLL-wt	Childhood T-cell ALL	ATCC	STR profiling 22/03/2016
K562	MLL-wt	CML	ATCC	STR profiling 22/03/2016
Hal-01	MLL-wt	ALL	A Thomas, Dana-Farber Cancer Institute, Massachusetts, USA	STR profiling 22/03/2016
UOC-B1	MLL-wt	Promyelocytic leukemia	A Thomas, Dana-Farber Cancer Institute, Massachusetts, USA	STR profiling 22/03/2016
NB4	MLL-wt	Acute promyelocytic leukemia	ATCC	STR profiling 22/03/2016
Calu-6	MLL-wt	Lung carcinoma	ATCC	STR profiling 22/03/2016
MCF-7	MLL-wt	Breast adenocarcinoma	ATCC	STR profiling 22/03/2016
HeLa	MLL-wt	Adenocarcinoma	ATCC	STR profiling 31/07/2014
BE(2)-C	MLL-wt	Neuroblastoma	J Biedler, Memorial Sloan-Kettering Cancer Centre, NY, USA	STR profiling 04/12/2012
HSF	MLL-wt	Primary skin fibroblast cells	ATCC	STR profiling 22/03/2016
MRC5	MLL-wt	Lung fibroblast	ATCC	STR profiling 04/02/2014
MCF10a	MLL-wt	Epithelial cell	ATCC	STR profiling 22/03/2016

a Method and date of authentication

b Cells up to ten passages from the original stock were used for experiments

### Small molecules

The chemical small molecule library consisting of 34,000 diverse compounds (DIVERSet) used for phenotypic screening as well as CCI-007 were purchased from Chembridge Corporation (San Diego, California, USA).

### Alamar blue cytotoxicity assay

Cells were seeded in duplicate wells at appropriate densities and incubated 24h at 37°C 5% CO_2_ prior to addition of test compounds (diluted in DMSO) or DMSO as vehicle control. After drug treatment for 72h (37°C, 5% CO_2_), Alamar Blue (10% (v/v)) (Sigma-Aldrich, Castle Hill, New South Wales, Australia) was added to assess cell viability. Absorbance was measured 5h after addition of Alamar Blue using a Benchmark Plus microplate reader (Bio-Rad, Gladesville, New South Wales, Australia) (570nm-590nm). Raw absorbance readings were used to generate dose-response curves. Half maximum inhibitory concentration (IC_50_) values for each compound were calculated by Fit Spline (Point-to-Point) analysis using GraphPad Prism 6 software.

### Apoptosis detection using Annexin V/7-aminoactinomycin D staining by flow cytometry

Treated cells were harvested by centrifugation, washed with PBS, stained with Annexin V and 7-Aminoactinomycin D (7-AAD) according to supplier specifications (BD Biosciences, North Ryde, New South Wales, Australia) and analysed on a BD FACSCanto Flow Cytometer (BD Biosciences). Data were analysed using FlowJo software (Treestar, Ashland, Orlando, USA) to determine apoptotic population distributions. To analyze the effect of caspase inhibition, cells were pre-treated with 10 μM pan-caspase inhibitor Q-VD-OPh (Sigma-Aldrich) for 2h.

### Analysis of mitochondrial depolarization by JC-1 flow cytometry

Treated cells were harvested by centrifugation, washed and incubated with JC-1 diluted in PBS (10 μg/ml) (Life Technologies) for 15 min at 37°C. Cells were transferred to FACS tubes and samples were run on a BD FACSCalibur Flow Cytometer (BD Biosciences). Data were analysed using FlowJo software (Treestar, Ashland, Orlando, USA) to determine depolarized and non-polarized cell populations.

### Cell cycle analysis using propidium iodide stain with flow cytometry

Treated cells were harvested by centrifugation, washed and kept in 70% chilled ethanol until subsequent analysis, at which point cell pellets were resuspended in Propidium Iodide (Sigma-Aldrich) diluted in PBS according to supplier specifications. Samples were protected from light and incubated for 10 minutes before transfer to FACS tubes via a 40 μm cell strainer (Corning, Pennant Hills, New South Wales, Australia). Samples were run on a BD FACSCalibur Flow Cytometer (BD Biosciences) and data were analysed with ModFit LT 3.0 (BD Biosciences).

### Quantitative real-time RT-PCR

RNA was extracted from treated cells using the RNeasy mini kit (Qiagen, Chadstone Centre, Victoria, Australia) according to the manufacturer's recommendations. RNA concentration and purity were determined using the NanoDrop 2000 Spectrophotometer (Thermo Scientific, Scoresby, Victoria, Australia). 0.5 to 2 μg mRNA was reverse transcribed in a reaction mix containing 10U/μL Moloney Murine Leukemia Virus reverse transcriptase (Life Technologies), 5 ng/μL Random Primers (Life Technologies), 1x first strand buffer (Life Technologies), 5 mM DTT (Life Technologies), 1 U/μL RNasin ribonucleotide inhibitor (Promega, Sydney, Australia) and 500 μM dNTP mix (Promega) for 1h at 37°C followed by addition of water to a final concentration of 40 ng/μL cDNA. Quantitative real-time RT-PCR (qRT-PCR) reactions contained 40 ng cDNA, 1x KAPA Probe Fast Mastermix (Geneworks, Thebarton, South Australia, Australia), 1x labelled primer and probe set (Sigma-Aldrich) or 1x TaqMan Probe-Primer set (Applied Biosystems, Life Technologies, Scoresby, Victoria, Australia). The following primers were purchased from Sigma-Aldrich: *HOXA9* Forward primer: 5′GAC AAG CCC CCC ATC GAT 3′; *HOXA9* Reverse primer: 5′ GAG TGG AGC GCG CAT GA 3′; *MEIS1* Forward primer: 5′ TCG CGC AGA AAA ACC TCT ATT 3′; *MEIS1* Reverse primer: 5′ TTG TCA CAT AAT TCG TGT ACC 3′. Predesigned TaqMan probe-primer sets for *CMYC* (Hs99999003_m1), *BCL2* (Hs00608023_m1) and *GUSB* (4326320E), *HPRT* (4326321E), *B2M* (4326319E) and *PGK1* (4326318E) as housekeeping genes were purchased from Applied Biosystems (Life Technologies). Each sample was run in duplicate or triplicate with an ABI7900HT Fast Real-Time PCR System (Applied Biosystems). Data analysis was performed using SDS2.3 software (Applied Biosystems). Relative expression was calculated by the ΔΔCT method and normalization against housekeeping genes and expressed relative to DMSO vehicle control.

### Sodium dodecyl sulphate polyacrylamide gel electrophoresis (SDS-PAGE) and immunoblotting

Cell pellets were lysed using 50 μL RIPA Buffer (Sigma-Aldrich) mixed with PhosSTOP phosphatase inhibitor (Roche, Dee Why, New South Wales, Australia) and Complete Protease Inhibitor (Roche) according to supplier specifications. Protein extract concentrations were determined using a bicinchoninic acid (BCA) assay kit (Pierce, Life Technologies, Scoresby, Victoria, Australia). 10 to 25 μg protein was denatured, loaded into pre-cast SDS-PAGE gels (Bio-Rad) and run. Proteins were transferred to polyvinylidene difluoride membranes (Merck Millipore, Bayswater, Victoria, Australia) and membranes were blocked with 5% non-fat dairy milk (NFDM) in Tris-Buffered Saline (TBS) containing 0.05% Tween-20 (TBS-T) for 1h before incubation with primary antibodies (rabbit anti-HoxA9 (1:2000, Merck Millipore, Bayswater, Victoria, Australia), rabbit anti-Meis1 (1:10,000 Abcam, Melbourne, Victoria, Australia), rabbit anti-cMyc (1:1000, Cell Signaling Technology, Danvers, Massachusetts, USA), rabbit anti-Bcl2 (1:1000, Cell Signaling Technology), rabbit anti-cleaved PARP (Cell Signaling Technology), rabbit anti-cleaved caspase-3 (Cell Signaling Technology), rabbit anti-actin (1:5,000 Sigma-Aldrich). Primary antibodies were diluted in TBS-T 5% NFDM and incubated with membranes overnight at 4°C. Membranes were washed with TBS-T and incubated with secondary goat anti-rabbit polyclonal (1:10,000, Life Technologies) in TBS-T 5% NFDM for 1h at room temperature. After washing with TBS-T and subsequently PBS, membranes were incubated with Pierce ECL western blotting substrate (Life Technologies) for 5 minutes and chemiluminescence was detected using Super HR-T30 medical X-ray film (Fujifilm, Sydney, New South Wales, Australia). Films were developed and fixed using an Okamoto X3 film processor (Lomaen Medical, Isando, South Africa). Where necessary, membranes were stripped of antibodies using Restore PLUS stripping buffer (Life Technologies) for 10 minutes at room temperature on a fast rocker, washed with PBS followed by washing with TBS-T, then re-blocked in TBS-T with 5% NFDM for 1h at room temperature. Films were scanned and analysed via semi-quantitative densitometry using ImageJ (http://rsb.info.nih.gov/ij/).

### Generation of CCI-007 resistant cell lines

CCI-007 resistant PER-485 cells were generated by culturing the parental PER-485 cell line in culture medium containing CCI-007 at concentrations above IC_90_ for 48h periods. Cells were collected by centrifugation and viability was assessed by trypan blue staining and cell counting. Cells were subsequently allowed to recover in culture medium without CCI-007. The cycles of exposure to these high concentrations of CCI-007 followed by recovery were repeated until at least 90% of the cells were viable after the 48h treatment. Alamar blue cytotoxicity assays were performed to verify the level of resistance. Two CCI-007-resistant pools, p1-12 and p1-15 were generated after more than twelve selection cycles.

### Immunofluorescence

Treated cells were deposited onto slides through a cytofunnel using a Shandon Cytospin for 3 minutes at 150g. Cells were air-dried, fixed with 4% paraformaldehyde for 10 minutes at room temperature and washed 3 times with PBS followed by a 0.2% TritonX-100 wash. Slides were blocked with 10% donkey serum (Sigma-Aldrich) in PBS for 10 minutes. Primary anti-HoxA9 (rabbit, Upstate Biotechnology, Bayswater, Victoria, Australia) and anti-Meis1/2/3 (mouse, Upstate Biotechnology) antibodies were added in 5% donkey serum for 2h at 37°C, diluted 1:50 and 1:75, respectively. After washing, slides were incubated with secondary antibody (Cy3-conjugated anti-mouse or anti-rabbit, 1:1000, Life Technologies) in 5% donkey serum for 1h at 37°C. After washing in PBS, cells were rinsed in distilled water followed by DAPI nuclear staining (Life Technologies). Cover slips were mounted and sealed and staining was visualized using a Zeiss Axiovert 100 fluorescence microscope (Carl Zeiss, North Ryde, New South Wales, Australia).

### Microarray and data analysis

PER-485 cells were seeded and treated with 5 μM CCI-007 or vehicle control for 3h. RNA was isolated and used for microarray gene profiling using the Illumina HumanHT arrays (Ramaciotti Centre for Genomics, University of New South Wales, Sydney, Australia). Samples were prepared in triplicate and tested in three independent experiments. Data were processed in R platform (https://www.r-project.org/), using Bioconductor packages *lumi* to normalize the microarray and *limma* to generate gene lists of differentially expressed genes in DMSO versus CCI-007 treated PER-485 cells. To determine whether genes overexpressed in MLL-r leukemia were downregulated in CCI-007-treated PER-485 and U937 cells, Gene Set Enrichment Analysis (http://www.broadinstitute.org/gsea/index.jsp) was performed with a False Discovery Rate (FDR) below 0.25 considered to be significant.

### Statistical analysis

Statistical analyses were performed with GraphPad Prism6 Software. Statistical significance of mean differences between groups was determined by performing ANOVA or Student's t-test with following significance levels: *, p<0.05; **, p<0.01; ***, p<0.001; ****, p<0.0001.

Principal Component Analysis on gene expression data obtained by quantitative real-time RT-PCR was performed in R using the prcomp() function from the built-in R *stats* package with the autoplot() function from *ggfortify* CRAN package for data visualization.

## SUPPLEMENTARY FIGURES


