# Supplementary material for: CCI-007, a novel small molecule with cytotoxic activity against infant leukemia with MLL rearrangements
Source: Oncotarget. 2016 Jun 14;7(29):46067–87. doi: 10.18632/oncotarget.10022 (PMC5216782; doi:10.18632/oncotarget.10022)
Supplement: Supplementary file 1 [file oncotarget-07-46067-s001.pdf]

CCI-007, a novel small molecule with cytotoxic activity against infant leukemia with *MLL* rearrangements

SUPPLEMENTARY FIGURES

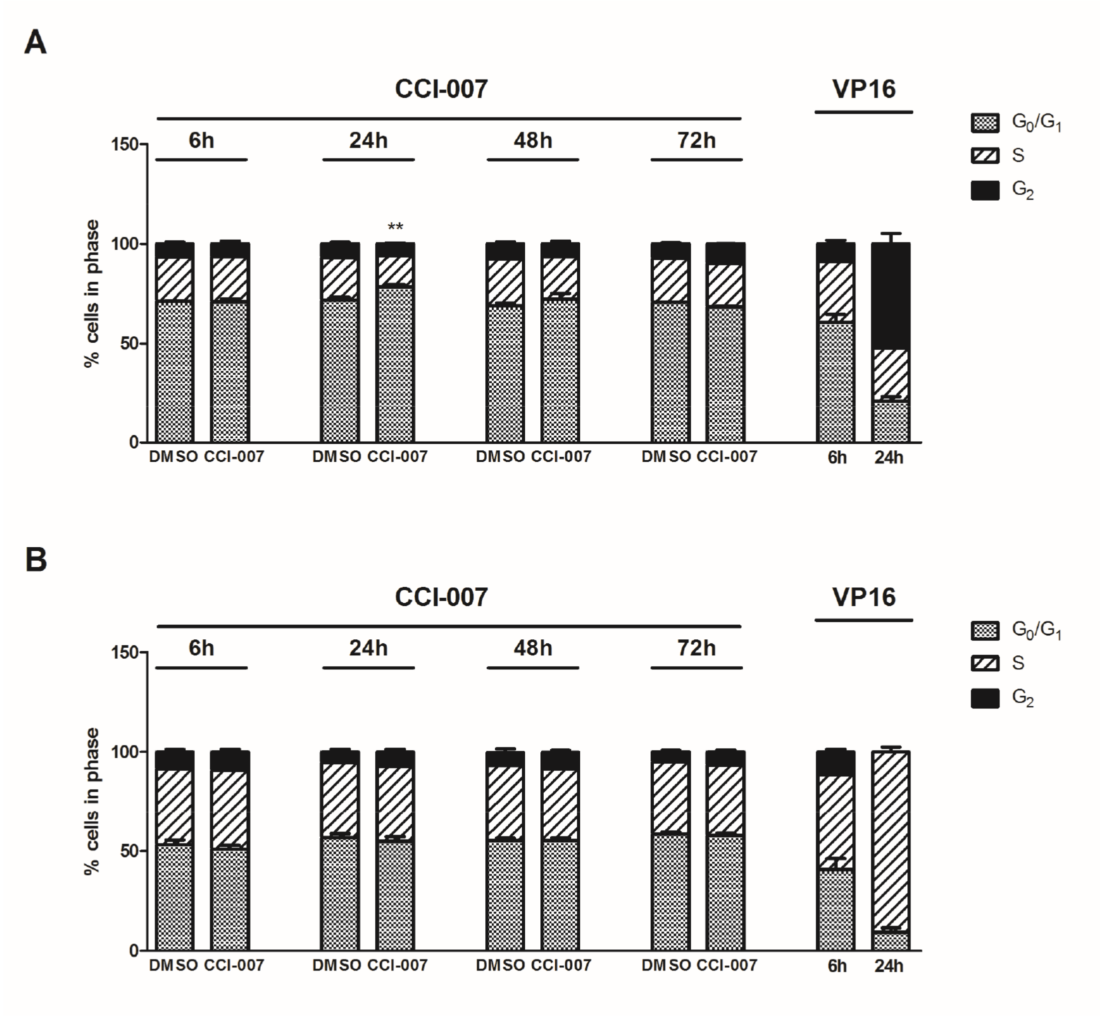

Supplementary Figure S1: Effect of CCI-007 treatment on cell cycle progression. Cell cycle analysis was performed on CCI-007 sensitive PER-485 cells **A.** and CCI-007 resistant MLL-wt REH cells **B.** treated with 5 µM CCI-007, vehicle control or 0.4 µg/ml VP16 (as a positive control for cell cycle block) for up to 72h using propidium iodide staining followed by flow cytometry. The percentages of cells in G_0/G_1, S and G_2 cell cycle phases are shown in the bar graphs representing mean ± SE of 3 independent experiments. Mean percentages of cells per phase between treatments were compared by t-tests. **, P<0.01.

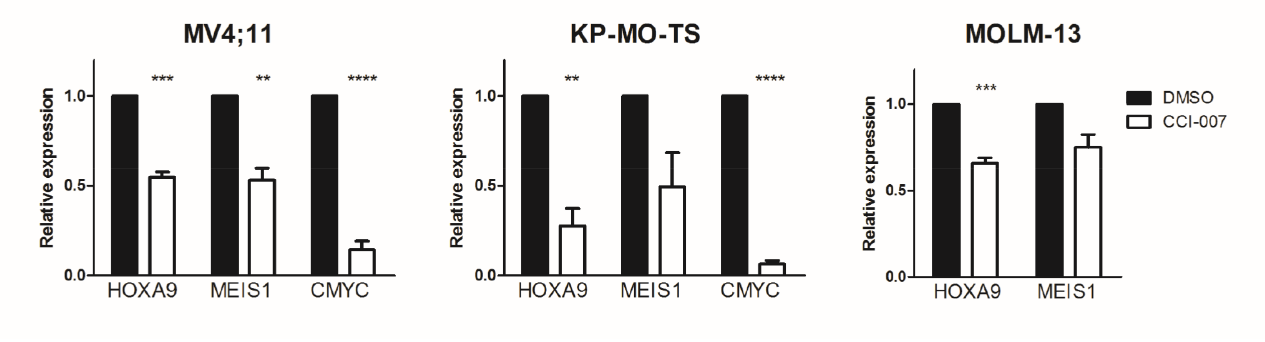

Supplementary Figure S2: CCI-007 treatment lowers *HOXA9, MEIS1* and *CMYC* mRNA levels in CCI-007 sensitive cell lines MV4;11, KP-MO-TS and MOLM-13. Effect of CCI-007 treatment on *HOXA9*, *MEIS1* and *CMYC* mRNA levels in sensitive MV4;11 (MLL-AF4), MOLM-13 (MLL-AF9) and KP-MO-TS (CALM-AF10) cells. Cells were treated with 5 µM CCI-007 (MV4;11 and KP-MO-TS) or 10 µM CCI-007 (MOLM-13) for 3h. mRNA levels were assayed by quantitative real-time RT-PCR and relative expressions were calculated using the ΔΔCt method. Gene expressions were normalized against housekeeping genes and expressed relative to DMSO vehicle control. Each data point represents the mean ± SE of at least 3 independent experiments. Mean relative expressions between treatment groups were compared by t-tests. **, P<0.01; ***, P<0.001; ****, P<0.0001.

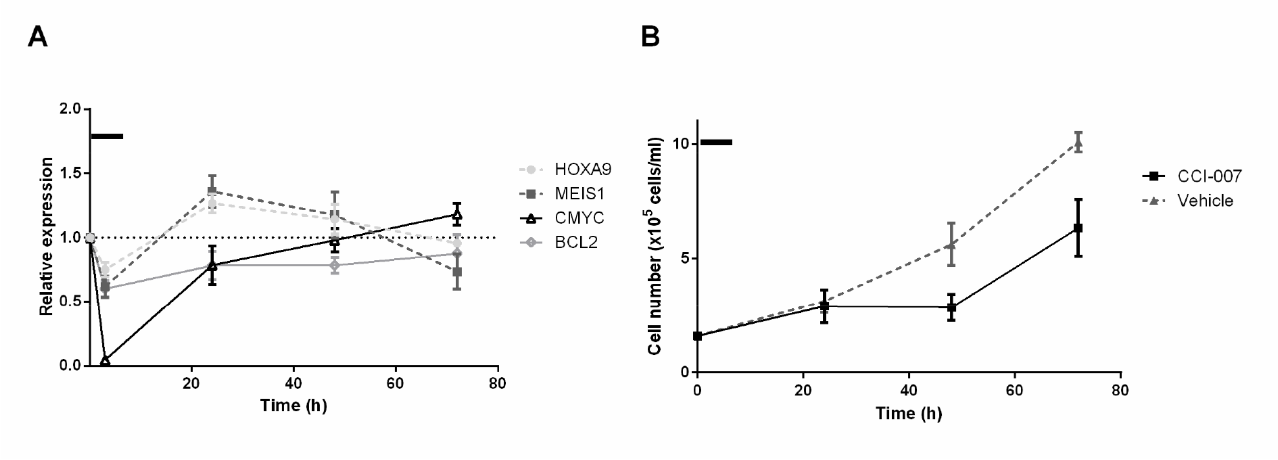

Supplementary Figure S3: MLL target gene expression levels and cell viability recover in parallel upon removal of CCI-007. PER-485 cells were treated transiently with CCI-007 or vehicle for 6h (black bar) after which compound was removed, cells were thoroughly washed and incubated in culture medium without compound. **A.** Messenger RNA levels of *HOXA9*, *MEIS1*, *CMYC* and *BCL2* were assayed by quantitative real-time RT-PCR and relative expressions were calculated using the ΔΔCt method. Gene expressions were normalized against housekeeping genes and expressed relative to DMSO vehicle control. **B.** Living cells were counted by trypan blue exclusion. Each data point represents the mean ± SE of at least 3 independent experiments.

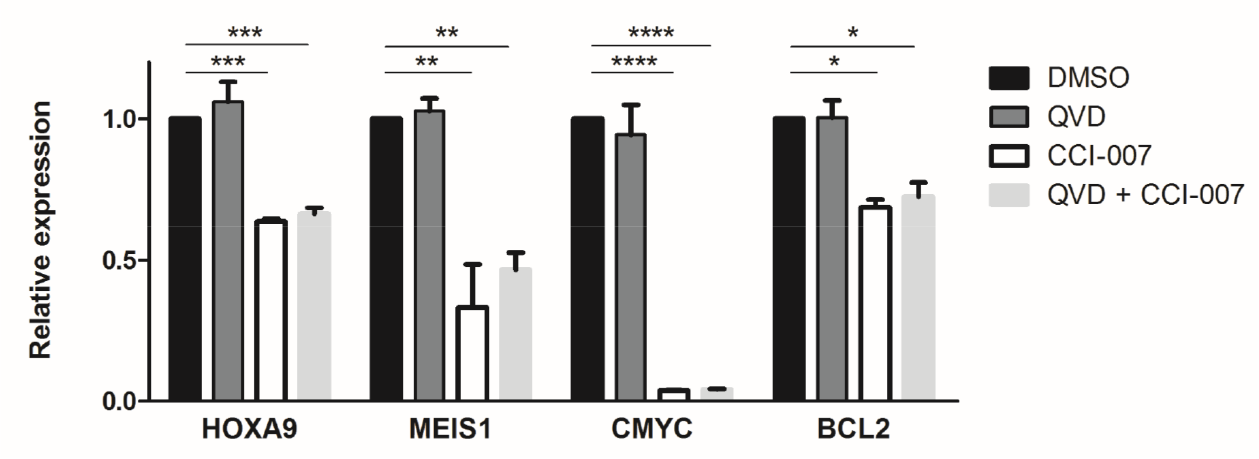

Supplementary Figure S4: CCI-007 decreases mRNA expression of MLL target genes independent from caspase-dependent apoptosis. CCI-007 treatment induces a reduction in MLL target gene mRNA levels when caspase-dependent apoptosis is inhibited. PER-485 were pre-treated with 10 µM pan-caspase inhibitor Q-VD-OPh for 2h prior to treatment with 5 µM CCI-007 for 3h. mRNA levels were assayed by quantitative real-time RT-PCR and relative expressions were calculated using the ΔΔCt method. Gene expressions were normalized against housekeeping genes and expressed relative to DMSO vehicle control. Samples were run in triplicate in each assay. Each data point represents the mean ± SE of 3 experiments. ANOVA was performed to compare mean relative expressions of genes between treatment groups. *, $P<0.05$; **, $P<0.01$; ***, $P<0.001$; ****, $P<0.0001$.

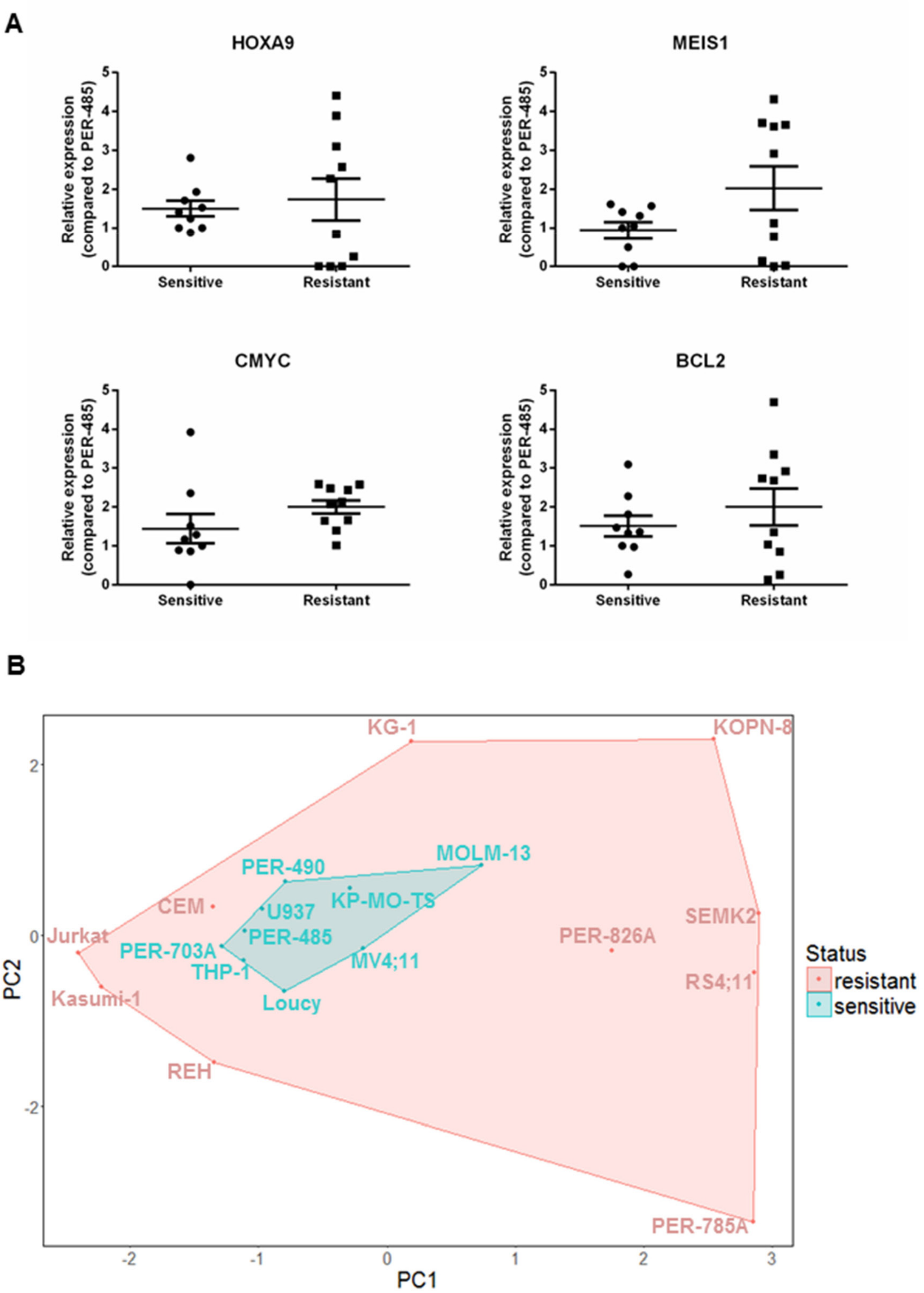

Supplementary Figure S5: Baseline gene expression levels of *HOXA9*, *MEIS1*, *CMYC* and *BCL2* do not significantly differ between CCI-007 sensitive and CCI-007 resistant leukemia cell lines within the broad leukemia cell line panel. CCI-007 sensitive (n=9) and CCI-007 resistant (n=10) leukemia cell lines (sensitivity and resistance as defined by IC_{50} as listed in Table 1) were harvested in the exponential growth phase. mRNA levels were assayed by quantitative real-time RT-PCR and relative expressions were calculated using the ΔΔCt method. Gene expressions were normalized against housekeeping genes and expressed relative to PER-485 cells. **A.** Each data point represents the mean ± SE of at least 3 independent experiments. Mean relative expressions between groups were compared by t-tests. **B.** Principal Component Analysis on relative expression data obtained by quantitative real-time RT-PCR for the broad leukemia cell line panel does not result in segregation of cells into separate clusters based on CCI-007 sensitivity.
